# Systemic Immune-Inflammation Index and Unconjugated Bilirubin Levels Are Associated with Pseudoexfoliation Syndrome

**DOI:** 10.18502/jovr.v20.17022

**Published:** 2025-12-08

**Authors:** Rafiye Nur Abay, Zeynep Katipoğlu

**Affiliations:** ^1^Kırşehir Ahievran Training and Research Hospital, Ophthalmology Department, Kırşehir, Turkey; ^2^Balıkesir Atatürk City Hospital, Department of Ophthalmology, Altıeylül, Balıkesir, Turkey

**Keywords:** Neutrophil-to-Lymphocyte Ratio, Pseudoexfoliation, Systemic Immune Inflammation Index, Unconjugated Bilirubin

## Abstract

**Purpose:**

To evaluate the systemic immune-inflammation index (SII) and unconjugated bilirubin levels in patients with pseudoexfoliation syndrome (PEX) and compare them with those of healthy individuals (controls).

**Methods:**

The PEX group (*n* = 48) and the control group (*n* = 88) were retrospectively evaluated. The results of ophthalmic examinations and routine laboratory parameters were analyzed, and the SII was calculated as follows: SII = platelet count × (neutrophil/lymphocyte).

**Results:**

The neutrophil-to-lymphocyte ratio (NLR) (*P *= 0.04) and SII (*P *= 0.03) were higher, and unconjugated bilirubin levels (*P* = 0.03) were significantly lower in the PEX group than in the control group. Univariate regression analysis showed that the unconjugated bilirubin levels (OR = 0.26; 95% CI, 0.07 to 0.95; *P* = 0.04) and SII (OR = 1.01; 95% CI, 1.00 to 1.01; *P *= 0.04) were correlated with PEX. Multivariate logistic regression analysis revealed that the correlation between bilirubin levels (OR = 0.02; CI, 0.01 to 0.25; *P *= 0.002) and SII (OR = 1.01; CI, 1.00 to 1.02; *P *= 0.001) remained statistically significant when adjusted for age, gender, and Charlson comorbidity index score.

**Conclusion:**

High SII and low bilirubin values in patients with PEX were found to be correlated.

##  INTRODUCTION 

The age-related disease known as pseudoexfoliation syndrome (PEX) is typified by the production and accumulation of extracellular material in several parts of the eye, particularly in the ciliary body, lens, capsule, zonules, and pupillary rim.^[1]^ PEX is the most frequently recognized cause of glaucoma,^[2]^ and its pathogenesis remains unclear, despite its high prevalence and clinical importance.^[3]^

PEX is both an ocular disease and a systemic one.^[4]^ Elevated ocular inflammation, endothelial dysfunction, and oxidative stress are possible leading causes for the condition.^[5]^ A growing body of research suggests that oxidative stress is an important variable in the pathophysiology of PEX.^[6,7]^ Cumurcu et al showed that PEX decreases total antioxidant capacity (TAC) and increases oxidative stress.^[8]^

Endogenously produced bilirubin has been shown to have significant antioxidant properties for scavenging peroxyl radicals. This property of bilirubin inhibits lipid oxidation.^[9]^ Several studies have investigated the antioxidant and anti-inflammatory properties of bilirubin. Akboğa et al reported that total serum bilirubin levels are inversely linked to both direct and indirect indicators of inflammation (C-reactive protein and neutrophil-to-lymphocyte ratio [NLR]) and the degree of coronary atherosclerosis in individuals with coronary artery disease.^[10]^

An inflammatory biomarker known as the systemic immune-inflammation index (SII) is proposed as a prognostic indicator for a variety of illnesses, including cancer and coronary artery disease.^[11,12,13,14]^ Ophthalmic studies have revealed a connection between SII and keratoconus, dry eye disease, and primary open-angle glaucoma.^[15,16,17]^

To our knowledge, no study has so far explored the importance of SII and bilirubin in patients with PEX. Thus, this study aimed to examine the connection between PEX, oxidative stress, and inflammation.

**Table 1:** Characteristics of the sample

**Variable**		**Total (** ***N*** ** = 136)**	**Pseudoexfolation syndrome **		***P*** **-value**
			**Positive (** ***N*** ** = 48)**	**Normal (** ***N*** ** = 88)**	
Demographics					
	Age (year), mean ± SD	68.0 ± 13.7	69.3 ± 7.6	67.3 ± 16.1	0.41
	Gender (Female), *n* (%)	81 (59.6)	28 (58.3)	53 (60.2)	0.83
	Charlson comorbidity index score, mean ± SD	1.5 ± 1.1	1.8 ± 1.2	1.1 ± 0.8	0.01
İnflammatory markers					
	NLR	2.2 ± 1.3	2.5 ± 1.3	1.9 ± 1.2	0.04
	Bilirubin (unconjugated)	0.6 ± 0.1	0.5 ± 0.1	0.6 ± 0.1	0.03
	Uric acid	5.6 ± 2.2	5.2 ± 1.7	5.8 ± 2.4	0.16
	SII	533 ± 449	643 ± 446	470 ± 441	0.03
Categorical variables are expressed in numbers (%) and determined by the chi-square test; Numerical variables with normal distribution are indicated as mean ± SD and determined by the t-test; NLR, neutrophil-lymphocyte ratio; SII, systemic immune-inflammation index.					

**Table 2:** Association of inflammatory markers with pseudoexfoliation syndrome

	**Unadjusted** * *		**Model** * *	
	**OR (95% CI)**	***P*** **-value**	**OR (95% CI)**	***P*** **-value**
NLR	1.37 (0.99 to 1.87)	0.05	–	–
Bilirubin (unconjugated)	0.26 (0.07 to 0.95)	0.04	0.02 (0.01 to 0.0.25)	0.002
Uric acid	0.87 (0.72 to 1.06)	0.17	–	–
SII	1.01 (1.00 to 1.01)	0.04	1.01 (1.00 to 1.02)	0.001
Model: adjusted for age, gender, Charlson comorbidity index score, and laboratory data; NLR, neutrophil-lymphocyte ratio; SII, systemic immune-inflammation index.				

**Figure 1:**
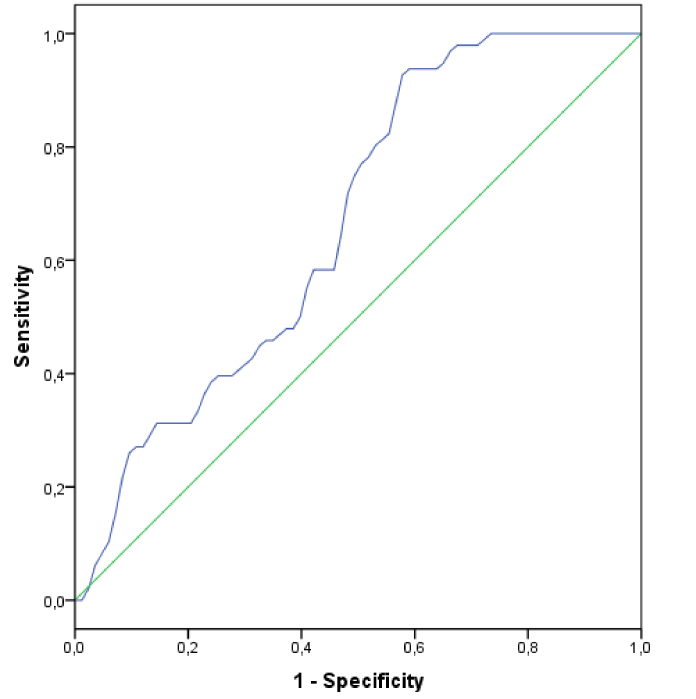
ROC curve analysis of SII in pseudoexfoliation syndrome.

##  METHODS

The PEX group consisted of 48 patients with PEX syndrome, aged 40–85 years, who were referred to the Ophthalmology Outpatient Clinic at Kırşehir Training and Research Hospital for eye examination between November 2022 and February 2023. On the other hand, the control group consisted of 88 people who had applied for a routine ophthalmic examination. Every patient underwent a comprehensive eye examination, which included a best-corrected visual acuity (BCVA) assessment, intraocular pressure measurement, anterior segment evaluation, and a fundus examination using a slit lamp biomicroscope. PEX diagnosis was made by observing white material deposits in the anterior segment, pupillary margin, or anterior lens capsule after pupil dilation.

Demographic data, ocular examination findings, and clinical and laboratory parameters of all participants were obtained from the information system and laboratory archives of Kırşehir Training and Research Hospital and then evaluated retrospectively. Approval for this study was obtained from the Clinical Research Ethics Committee of the Faculty of Medicine at Kırşehir Ahi Evran University (Turkey, approval number 2023-02/14). We conducted this work in accordance with the principles of the Declaration of Helsinki.

The exclusion criteria were eye diseases other than PEX syndrome previously diagnosed by an ophthalmologist, age younger than 18 years, advanced organ failure (liver, kidney), history of active infection, smoking, alcohol use, and impaired cognitive function.

Participants gave venous blood samples between 08:0 and 10:00 AM after 8 hours of fasting. The blood samples were then centrifuged at 4000 rpm for 10 minutes to separate serum and plasma samples. Biochemical parameters were measured using the Beckman Coulter AU5800 automated biochemical analyzer (Beckman Coulter Inc., Brea, CA, USA). The SII was calculated as the platelet count divided by the neutrophil/lymphocyte. Complete blood count values were measured using the Sysmex XN-1000 hematology analyzer (Sysmex Corporation, Kobe, Japan). The overall comorbidity burden was assessed using the Charlson Comorbidity Index (CCI) by assigning a weighted score to 17 possible comorbid conditions according to their 1-year relative mortality risk.^[18,19]^

### Statistical Analysis

IBM SPSS Statistics for Windows, version 24.0 (IBM Corporation, Armonk, NY, USA), was used for statistical analysis. The Kolmogorov-Smirnov test was used to assess whether the data followed a normal distribution. Normally distributed variables were presented as mean ± standard deviation, and non-normally distributed variables as median. The Student *t*-test or Mann-Whitney U test was run to investigate the continuous variables. The category data were compared using the chi-square test. Univariate and multivariate logistic regression analyses were conducted to identify the independent determinants of PEX. A model incorporating comorbidities, age, and gender was used to examine the independent relationship between PEX and inflammatory markers. With the SII as the test variable and PEX as the state variable, the receiver operating characteristic (ROC) curve was formulated, and the diagnostic value of the SII was determined based on the area under the curve (AUC). The ideal cut-off value was determined using the Youden index, and *P* < 0.05 was deemed statistically significant.

##  RESULTS

A total of 130 participants were included (mean age: 68.0 ± 13.7 years, male: 59.6%). Table 1 shows the demographic data and laboratory parameters of the entire study population.

The PEX and the control groups did not differ significantly by gender (*P *= 0.83) or age (*P* = 0.41). The PEX group had higher CCIs (*P* = 0.01). Upon analyzing laboratory parameters, no statistically significant difference was found in uric acid levels between the two groups (*P* > 0.05). However, the NLR and SII values in the PEX group were significantly higher (*P* < 0.05). Conversely, the levels of unconjugated bilirubin were significantly lower in the PEX group compared to the control group. (*P *= 0.04)

The univariate regression analysis revealed that bilirubin levels (OR = 0.26; CI, 0.07 to 0.95; *P* = 0.04) and SII (OR = 1.01; CI, 1.00 to 1.01; *P* = 0.04) were associated with PEX. This association between bilirubin (OR = 0.02; CI, 0.01 to 0.25; *P *= 0.002) and SII (OR = 1.01; CI, 1.00 to 1.02; *P* = 0.001) and PEX maintained statistical significance after adjusting for gender, age, and CCI in the multivariate logistic regression analysis (model) [Table 2].

The ability of SII to predict PEX was assessed using the ROC curve analysis. The resulting AUC value is presented in Figure 1, revealing that SII is a statistically significant predictor of PEX. According to the Youden index, the SII cut-off value for PEX was 434.2 (AUC = 0.67, sensitivity = 60%, specificity = 60%; *P* = 0.002) [Figure 1].

##  DISCUSSION

In this study, NLR, SII, oxidative stress, and inflammatory markers were higher, while unconjugated bilirubin levels were lower, in the PEX group compared to the control group. Moreover, even after several confounding variables were taken into account, these markers were still significantly associated with the disease. Thus, inflammation and oxidative stress are significant factors in the etiopathogenesis of PEX. These markers can be readily calculated from basic blood parameters and used in daily practice. To the best of our knowledge, these findings are the first of their kind in the literature.

Genetic and nongenetic factors, including age, race, autoimmune diseases, viral infections, inflammation, oxidative stress, and vascular structure, are associated with the pathogenesis of PEX.^[20]^ Inflammatory biomarkers and antioxidant parameters are becoming important indicators for evaluating diseases. In recent years, peripheral cell counts and their combinations have been used as indicators of systemic and ocular inflammation.^[17]^ Studies have shown that immune and inflammatory responses are important in the development of atherosclerosis. The predictive efficacy of SII in cardiovascular diseases has been confirmed.^[21]^ In another study of ours, triglyceride-glucose (TyG) indices, which are considered an atherogenic indicator, were found to be notably higher in the PEX group compared to the control group.^[22]^ Xiao et al also reported that SII showed a linear positive correlation with the TyG index.^[23]^ In addition to cardiovascular diseases, SII is also correlated with inflammatory hepatic steatosis, autoimmune rheumatoid arthritis, and some cancers.^[24,25,26]^

Elbeyli et al showed that elevated SII was strongly associated with diabetic macular edema in patients with non-proliferative diabetic retinopathy, in which systemic and retinal inflammations play a role.^[27]^ Wang et al found that NLR and SII values were noticeably positively correlated with interleukin-6 (IL-6) levels in the aqueous humor of young patients with ischemic retinal vein occlusion. They also demonstrated that systemic inflammation was a major factor in the onset of disease in young patients.^[28]^ NLR has also been shown to increase in diseases such as dry eye, PEX, open-angle glaucoma, and keratoconus.^[15,17,20,29]^ The results of previous studies revealed that the SII, in which all three peripheral cells are examined, can be highly effective in understanding the etiopathogenesis of patients with PEX.

There are many studies on oxidative stress in patients with PEX. Cumurcu et al showed that the serum TAC was low and the total oxidant status was elevated in patients with PEX.^[8]^ Gartaganis et al reported that reactive oxygen species, such as glutathione, glutathione disulfide, and thiobarbituric acid, which cause free radical damage, are found to a higher extent in the aqueous humor of patients with PEX.^[6]^ Yilmaz et al noticed that serum vitamin C concentrations were substantially lower and malondialdehyde concentrations were significantly higher in these patients.^[30]^ Yildirim et al reported that the concentration of zinc, an antioxidant substance, was low in the lenses of PEX patients extracted during cataract surgery.^[31]^ Unconjugated bilirubin has been shown to have antioxidant effects; in our study, its level in the PEX group was lower than in the control group.

Uric acid is a product of purine metabolism in human plasma and is an important water-soluble antioxidant product.^[32]^ The literature shows that uric acid, which has antioxidant properties, is associated with cardiovascular diseases, hypertension, and diabetes mellitus.^[33,34,35]^ Studies show a significant inverse association between serum uric acid concentrations and the risk of glaucoma.^[32,36]^ Uric acid levels were reported to be considerably higher in one study of patients with PEX, although no statistically significant difference was observed in another investigation.^[37,38]^ Since there are mixed results in the literature regarding the levels of uric acid, we evaluated this variable in patients with PEX in the present study. In our study, uric acid levels were found to be lower in patients with PEX; however, this difference was not statistically significant. When the results were evaluated together with our study, no consensus was reached. Further clinical studies are needed to clarify the potential role of uric acid in diseases associated with oxidative stress.

This study had some limitations, including its retrospective, single-center design and the small number of participants. Therefore, further multicenter clinical studies with larger patient groups are required.

In summary, this study demonstrated a significant association between PEX and systemic inflammatory markers, as well as the antioxidant unconjugated bilirubin. These are the first findings of this kind in the literature, and they shed light on the pathogenesis of PEX. This study may also guide researchers in diagnosing and understanding the progression of the disease.

##  Financial Support and Sponsorship

None.

##  Conflicts of Interest

None.
